# RCC-Supporter: supporting renal cell carcinoma treatment decision-making using machine learning

**DOI:** 10.1186/s12911-024-02660-7

**Published:** 2024-09-16

**Authors:** Won Hoon Song, Meeyoung Park

**Affiliations:** 1 Department of Urology, Pusan National University School of Medicine, Yangsan, Republic of Korea; 2 Department of Urology, Pusan National University Yangsan Hospital, Yangsan, Republic of Korea; 3 Department of Computer Engineering, Kyungnam University, 7, Gyeongnamdaehak-ro, Masanhappo-gu, Changwon-si, 51767 Gyeongsangnam-do Republic of Korea

**Keywords:** Renal cell carcinoma, Medical big data, Clinical decision support system, Machine learning, Common data model

## Abstract

**Background:**

The population diagnosed with renal cell carcinoma, especially in Asia, represents 36.6% of global cases, with the incidence rate of renal cell carcinoma in Korea steadily increasing annually. However, treatment options for renal cell carcinoma are diverse, depending on clinical stage and histologic characteristics. Hence, this study aims to develop a machine learning based clinical decision-support system that recommends personalized treatment tailored to the individual health condition of each patient.

**Results:**

We reviewed the real-world medical data of 1,867 participants diagnosed with renal cell carcinoma between November 2008 and June 2021 at the Pusan National University Yangsan Hospital in South Korea. Data were manually divided into a follow-up group where the patients did not undergo surgery or chemotherapy (Surveillance), a group where the patients underwent surgery (Surgery), and a group where the patients received chemotherapy before or after surgery (Chemotherapy). Feature selection was conducted to identify the significant clinical factors influencing renal cell carcinoma treatment decisions from 2,058 features. These features included subsets of 20, 50, 75, 100, and 150, as well as the complete set and an additional 50 expert-selected features. We applied representative machine learning algorithms, namely Decision Tree, Random Forest, and Gradient Boosting Machine (GBM). We analyzed the performance of three applied machine learning algorithms, among which the GBM algorithm achieved an accuracy score of 95% (95% CI, 92–98%) for the 100 and 150 feature sets. The GBM algorithm using 100 and 150 features achieved better performance than the algorithm using features selected by clinical experts (93%, 95% CI 89–97%).

**Conclusions:**

We developed a preliminary personalized treatment decision-support system (TDSS) called “RCC-Supporter” by applying machine learning (ML) algorithms to determine personalized treatment for the various clinical situations of RCC patients. Our results demonstrate the feasibility of using machine learning-based clinical decision support systems for treatment decisions in real clinical settings.

## Background

Cancer can occur in the renal parenchyma. Histologically, more than 90% of kidney cancer cases are attributed to renal cell carcinoma (RCC), of which 70% are clear cell RCC, 10–15% are papillary RCC, and 5% are chromophobe RCC [1]. In 2020, approximately 431,288 individuals worldwide were newly diagnosed with kidney cancer. The population diagnosed with RCC in Asia accounts for 36.6% of all RCC cases worldwide [2], and the incidence rate of RCC in South Korea is continuously increasing every year [3]. In general, synchronous metastasis, which is generally defined as distant metastasis that occurs with, or within the three-month interval of the diagnosis, of the primary cancer, occurred in one-third of the newly diagnosed RCC patients and 20–40% of local RCC patients [4, 5]. Furthermore, the 10-year survival rate of patients with metastatic RCC was less than 5%, which is considered as a significantly malignant carcinoma [5–7].

A diverse range of treatment methodologies are available for RCC, such as such as active surveillance, partial nephrectomy, radical nephrectomy, chemotherapy, and cytoreductive nephrectomy combined with chemotherapy, depending on the clinical stage and histologic characteristics. Although clinical guidelines suggest standard treatment methods, in practice, different treatment methods are selected according to the various clinical conditions and characteristics of each patient [8]. Therefore, patients are treated with different surgical methods and chemotherapeutic agents depending on their condition.

In large hospitals in South Korea, the ratio of patients to clinicians is enormous, resulting in significantly short clinical practice hours per person. In this scenario, clinicians face difficulty in explaining the various treatment methods to each patient so that he/she can make an informed decision about the appropriate one. Therefore, the development of a clinical decision-support system (CDSS) for clinicians and patients with personalized standards of examination and treatment is required. This can be achieved by implementing artificial intelligence (AI)-based automatic treatment protocols suitable for the health condition of each patient.

In the field of medical AI, research on the development of a CDSS that can be customized for a specific disease using various AI algorithms to implement precision medicine based on medical big data has been actively conducted [9–18]. However, in the case of kidney cancer, few studies have applied AI because of the heterogeneous format and unstructured nature of medical data, such as pathology reports or radiology data. To address this challenge, multi-institutional international joint research using a common data model (CDM) that standardizes medical terminology and database structures has been actively conducted [19, 20].

In this study, real-world medical data comprising both structured data, which were obtained using the CDM constructed at our hospital, and unstructured data, namely pathologic results and computed tomography (CT) readings, which are the most important data for RCC treatments, were collected. All data were preprocessed to construct an RCC-related standard large database that could be used for downstream analyses. The purpose of this study was to develop a preliminary personalized treatment decision-support system (TDSS) called “RCC-Supporter”, applying machine learning (ML) algorithms to determine personalized treatment customized for the various clinical situations of RCC patients. We believe that our research will help clinicians execute more precise decisions regarding the optimal treatment options for RCC in a clinical setting.

## Methods

### Study design

We conducted a retrospective observational study to evaluate the possibility of developing a decision-making support system for treating RCC, called “the RCC-Supporter,” based on the real-world data (RWD) obtained from a hospital. We reviewed the de-identified data of 1,876 participants diagnosed with RCC between November 2008 and June 2021 at the Pusan National University Yangsan Hospital (PNUYH) in South Korea. The Observational Medical Outcomes Partnership (OMOP) CDM is a medical data standard adopted by the Observational Health Data Sciences and Informatics consortium to systematically analyze data not only in South Korea but also in North America, Europe, and Asia [38]. The CDM standardizes diverse hospital data into a unified database format, enabling collaborative research across various medical institutions globally. We gathered structured data from the OMOP CDM in conjunction with unstructured data, such as radiology reports, from electronic health records (EHRs) to construct a standard RCC big database. Feature selection was performed using the random forest ML algorithm to identify the important factors that affect RCC treatment decisions. We then employed tree-based explainable ML and ensemble algorithms to formulate a refined RCC-Supporter. Finally, the performances of the algorithms were evaluated based on four metrics: precision, recall, F1-score, accuracy score, and ROC curve for the overall performance. This study was conducted in accordance with the Declaration of Helsinki, reported according to the Strengthening the Reporting of Observational Studies in Epidemiology statement, and approved by the Institutional Review Board (IRB) of PNUYH, which waived the requirement for an IRB review in this study (IRB No. L-2020-466, 2020/10/25). Patient consent was waived due to the data for this study being de-identified and based on longitudinal observational health data.

### Data preparation

Participants newly diagnosed with rcc during hospital visits were reviewed. we obtained the data of 1,867 individuals from the omop cdm, which were converted from ehrs obtained from 2008 to 2021 at pnuYH. As a tertiary referral hospital, PNUYH has recently converted RWD, such as EHRs, into OMOP CDM. Structured data of the participants were collected from OMOP CDM tables, including “Person” (demographic information), “Condition” (diseases diagnosed during hospital visits), “Measurement” (laboratory results), “Procedures” (surgery information), and “Drug” (prescription drug information). Unstructured data, such as pathology reports and CT readings, which are the most important data for deciding the cancer treatment methods, were directly extracted from the EHRs with the cooperation of the hospital information and computer systems management team. All data were de-identified before collection and were validated by clinical experts.

### Data preprocessing

We defined the “index date” as the date on which the participants were newly diagnosed with RCC during the first hospital visit. The information related to RCC was extracted from the OMOP CDM condition table based on the OMOP CDM disease concept codes corresponding to the 10th revision of the International Classification of Diseases (ICD-10) and Systematized Nomenclature of Medicine — Clinical Terms (SNOMED-CT).

We examined the demographic information, such as age and gender, on the index date. The laboratory test results, including creatinine, hemoglobin, and lactate dehydrogenase (LDH) values for a period of seven days before and after the index date, all prescription drugs, and procedure information after the index date were extracted. Pathology and CT results were obtained at the closest dates before and after the index date.

As numerical and categorical data were mixed in the collected data, preprocessing was performed to convert them to numeric data for downstream data analysis. The standard scaling method that assigns the values of 0 and 1 to the mean and standard deviation, respectively, was applied to all numerical data before further analysis. The pathology and radiology results, which are unstructured, common, and essential features for RCC treatment decisions, were extracted in consultation with clinical experts. Missing values in the data were replaced using the multivariate imputations by chained equations (MICE) package in the R software. Among the patient data, cases with more than 50% missing values and outliers outside the standard distribution of data were excluded.

### Machine learning analysis

**Fig. 1:**
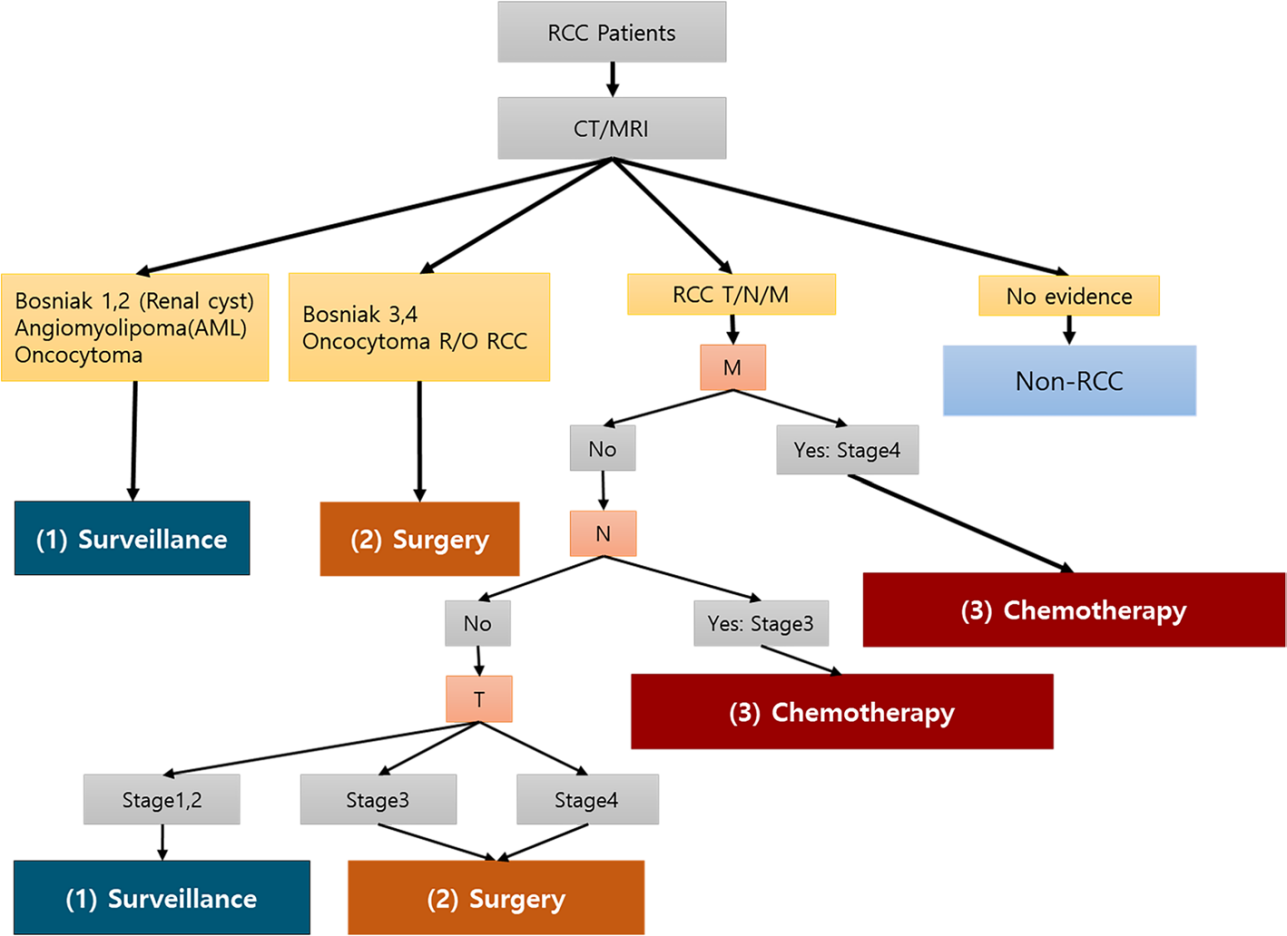
Grouping participants according to simplified treatment methods. Benign renal diseases such as Bosniak classification 1 and 2 of renal cyst, angiomyolipoma, and oncocytoma in the CT readings were classified as surveillance. Surgery is performed when RCC and Bosniak classification 3 and 4 of renal cyst with possible malignancy are suspected in the CT readings. In addition, according to the TNM stage, low stage was classified as surveillance or surgery, and high stage was classified as surgery or chemotherapy

The treatment methods were divided into three groups to determine the feasibility of the system and evaluate its performance. A urology clinician at PNUYH labeled the treatment methods based on the CT reading results, which is a critical criterion for all participants. Thus, the patients were divided into a follow-up group that did not receive surgery or chemotherapy (Group1-Surveillance), a group that underwent surgery (Group2-Surgery), and a group that received chemotherapy before or after surgery (Group3-Chemotherapy) (Fig. 1).

Based on the constructed standard RCC big data, ML algorithms were written to develop a TDSS. The algorithms extracted 2,062 features from the medical data (including the CDM data), which included the basic information of the patient, laboratory test information, and prescription drug information, in addition to pathology reports and CT readings extracted from the EHR. Feature selection using a random forest algorithm was performed to identify important features that affect the classification of renal cancer treatment methods. Additionally, after ranking by feature selection across the entire dataset, features ranked 20, 50, 75, 100, and 150 were selected as feature sets, respectively, to see how the number of features affects classification performance. Moreover, 50 features selected by clinicians (Expert50) were added to compare the feature selection method. Furthermore, to investigate the potential for improving accuracy, we combined selected features obtained from different methods and utilized them as inputs for machine learning. This process involved various combinations, including RF features with expert features, RF features with GBM features, GBM features with expert features, as well as combinations of GBM, RF, and expert features.

In this research, we chose an interpretable decision-tree model to generate treatment decisions that can be readily comprehended by both clinicians and patients in a clinical setting. We employed the random forest algorithm, an ensemble model based on decision tree principles known for its exceptional performance in ML tasks, in conjunction with the GBM, which is recognized for its ability to mitigate overfitting in datasets with imbalances. To validate the stability of machine learning models, nested cross-validation with stratified N-fold cross-validation techniques were used. Nested cross-validation is an approach to overcome the overfitting problem and to reduce the bias in performance evaluation [39]. Hyperparameters were optimized using GridSearchCV() function in Python. Finally, we evaluated the performance of ML algorithms. Our study can be regarded as a multi-class classification problem for classifying three categories. Therefore, we employed precision, recall, F1 score, and accuracy suitable for multi-class problems [40]. Additionally, ROC curves were generated to assess the overall performance of each algorithm for each feature set [41]. In this study, the classes to be predicted are three treatment methods: class 1- Surveillance, class 2- Surgery, and class 3- Chemotherapy. It can be considered as a multi-class classification problem to predict one of the three treatment methods. The task of generating a confusion matrix for multi-class classification involves binary classification for each class, resulting in separate counts of True Positive (TP), True Negative (TN), False Positive (FP), and False Negative (FN) for each class. That is, taking the Surveillance class as the reference, Surveillance becomes Positive while surgery and chemotherapy become Negative. Similarly, surgery and chemotherapy classes also generate their own TP, TN, FP, FN counts in this manner. Performance evaluation metrics such as precision, recall, F1-score, and accuracy were computed based on the calculated confusion matrix on the test set using the optimal set of hyperparameters. Furthermore, in binary classification, ROC curves plot Sensitivity against 1-specificity. However, in the case of multi-class classification, there are different methods to aggregate values calculated from each confusion matrix into a single value. One approach is the Micro Average method, where TP is calculated as the sum of TP for each class (e.g., TP for Surveillance + TP for Chemotherapy + TP for Surgery). Another method is the Macro Average method, where evaluation metrics are computed for each class separately, and then averaged to obtain an overall metric. We utilized the Macro Average method in our analysis.

## Results

### Schematic overview of the study

We identified 1,867 (63% male, 37% female) participants who were diagnosed with RCC between 2008 and 2021. Details of the number of participants, and their gender, and age of participants under the categories of Group 1 (Surveillance), Group 2 (Surgery), and Group 3 (Chemotherapy) are listed in Table 1. The age of the participants ranged between 17 and 88 years. Overall, 2,062 features of the RCC data were used.

**Table 1:** Distribution of study participants

	Group1 (Surveillance)	Group2 (Surgery)	Group3 (Chemotherapy)	Total
Number of participants	748 (40%)	893 (48%)	226 (12%)	1867 (100%)
Gender				
Male	432 (58%)	590 (66%)	163 (72%)	1185 (63%)
Female	316 (42%)	303 (34%)	63 (28%)	682 (37%)
Age (years)	57.36 ± 13.56	60.75 ± 12.90	64.37 ± 12.15	

### Data preprocessing

Due to the incomplete characteristics of the real-world data (RWD), we preprocessed the dataset without loss of any clinical information. Data imputation was applied if the features had missing values using predictive mean matching techniques in the multivariate imputations by chained equations (MICE) package provided in the R software [21]. After removing the outliers, the final number of features for modeling was 2,058. Table 2 summarizes the source and data types of the features extracted from the dataset. Then, we applied standard scaling method to improve the performance the ML algorithms.

**Table 2:** Data types of features

Features No.	Source	Data types
1–10	Basic demographic information	Category (yes: 1, no: 0), Numeric
11–42	CT report	Category (yes: 1, no: 0)
43–244	Measurement	Category (yes: 1, no: 0), Numeric
245–577	Procedure (Operation)	Category (yes: 1, no: 0)
578–2062	Drug	Category (yes: 1, no: 0)

### Feature selection

Feature selection was performed using the random forest algorithm to reduce the complexity of the model. Moreover, this also accounts for the fact that clinicians generally check fewer than 50 factors when making treatment decisions. Therefore, we identified the most effective number of features by testing the algorithms using 20, 50, 70, 100, and 150 features out of 2,058 features. Additionally, another 50 features (Expert50) were manually selected by the clinician to compare the results with those of the feature selection method. Among them, the common top ten features were selected by performing feature selection from 2,058 features (Table 3). These were consistent with the important factors that determine the treatment of RCC patients in practice. Among the selected features, the top five features were the diagnosis name (RCC), which represents the most important clinical diagnosis, and clinical stages where the overall stage is determined after the cancer is assigned a letter or number to describe the tumor (T), node (N), and metastasis (M) categories in the CT readings. Moreover, the 6th to 10th features were important diagnostic laboratory test information related to RCC in the clinical field. Most important feature was ‘RCC IN CT’. ‘RCC IN CT’ refers to the radiological diagnosis of kidney cancer in the CT readings. ‘T nan IN CT’ refers to the T (tumor size) stage itself, although the exact stage referred to in the CT readings is not mentioned. ‘N nan IN CT’ also refers to the N (metastasis of lymph node) stage, but the exact stage mentioned in the reading is unknown.

**Table 3:** Top 10 ranked features according to feature selection algorithm

Rank	Feature Importance	Data source
1	RCC IN CT	CT readings
2	T nan IN CT	
3	N nan IN CT	
4	N 0 IN CT	
5	T 1 IN CT	
6	Alkaline phosphatase	Lab test
7	Hemoglobin	
8	Red blood cell count	
9	Hematocrit	
10	Lactate dehydrogenase	

‘N 0 IN CT’ refers to the initial N stage without metastasis of lymph node in the CT readings. ‘T 1 IN CT’ refers to the early T stage in which the tumor size is 7 cm or less in the CT readings. In lab tests, alkaline phosphatase (ALP) is an enzyme present in the bile duct in hepatocytes and is rapidly elevated mainly in bile excretion disorders. In addition, it is present in most organs such as bones, placenta, and small intestine, so it can increase in diseases of those organs. Hemoglobin, Hematocrit, and red blood cell count are items in the complete blood count (CBC) test, which is the most basic blood test item in lab tests, and are items related to blood cells. Lactate dehydrogenase (LDH) is one of the enzymes that act when glucose is broken down and converted into energy. It is contained in many tissue cells, so when cells are destroyed, LDH in the blood increases. Blood LDH is often highly active in malignant tumors, liver diseases, heart diseases, blood diseases, etc., and is a useful test for screening these diseases.

### Machine learning algorithms

To develop our treatment decision support system, “RCC-Supporter”, we used an explainable decision tree (DT) algorithm and two ensemble algorithms, namely random forest (RF) and Gradient Boosting Machine (GBM). The decision tree is an algorithm that generates explainable classification rules by automatically identifying specific conditions by learning from data and creating a binary tree for prediction [22]. It is a significantly useful algorithm for non-ML experts to interpret the results. Therefore, this algorithm is useful for deriving results that can be intuitively understood by clinical experts and patients in the medical field. The random forest algorithm is an ensemble algorithm that generates multiple decision trees by applying a bagging method, and it subsequently aggregates the results for prediction [23]. It is the most popular ML algorithm because it reduces the risk of overfitting in limited data and can represent the importance of features with a classification indicator. The GBM algorithm applies a boosting method that gradually improves the errors by assigning weights to erroneously predicted data in imbalanced data [24–26]. We adopted these ensemble algorithms to reduce overfitting as our data were imbalanced as shown in Table 1.

To validate the stability of our machine learning models, we applied nested cross-validation (10 outer iterations and 3 inner iterations) using stratified 10-fold cross-validation techniques. As a result, each model underwent a training process 300 times. The models’ optimal hyperparameters were tuned using the GridSearchCV function in the Scikit-Learn module of Python [27]. As shown in Table 4, for the decision tree and random forest algorithms, the depth of the model (max_depth) and the minimum number of samples (min_samples_split) for each node in the tree were adjusted to prevent overfitting. The random forest method also considered the number of classifiers to be created (n_estimators). For the GBM, the depth of the model (max_depth), number of classifiers (n_estimators), and learning rate applied during each training iteration were adjusted.

**Table 4:** Best tuned hyperparameters for the machine learning models

Model	Parameters	Hyperparameter values for each feature set						
		20	50	75	100	150	All	Export50
Decision Tree	max_depth	4	4	6	6	6	6	5
	min_samples_split	13	22	21	21	20	21	17
Random Forest	n_estimators	300	200	250	250	200	300	250
	max_depth	13	14	15	14	15	13	13
	min_samples_split	9	10	9	8	8	8	10
GBM	n_estimators	200	250	250	300	300	300	300
	max_depth	7	7	5	5	5	5	7
	learning_rate	0.05	0.1	0.1	0.01	0.05	0.05	0.01

### Predictive performance

**Fig. 2:**
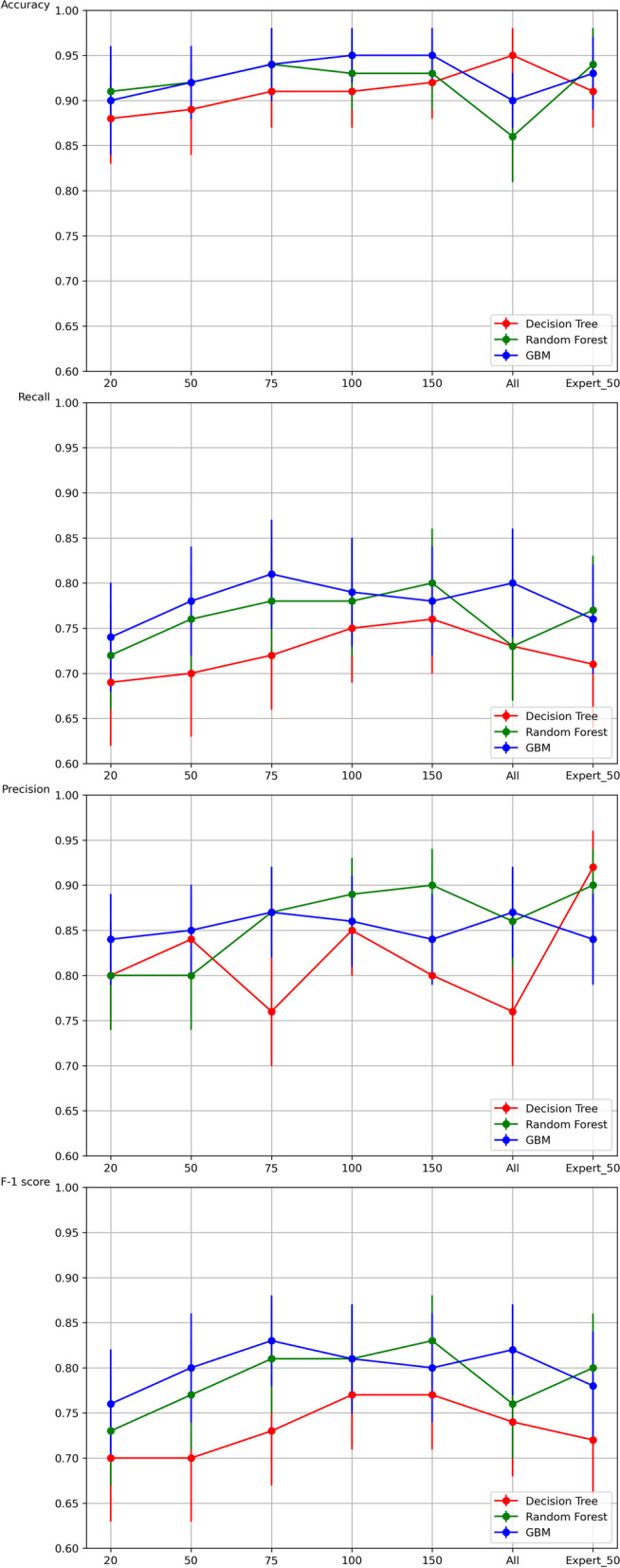
Comparison of the decision tree, random forest and gradient boosting machine performance after feature selection. The performances of the three machine learning algorithms based on seven different feature sets, namely with 20, 50, 75, 100, 150, all, and 50 expert-selected (Expert50) feature sets

Table 5; Fig. 2 summarize the performances of the three machine learning algorithms based on seven different feature sets: 20, 50, 75, 100, 150, all, and 50 expert-selected (Expert50) features. Performance evaluation metrics such as precision, recall, F1-score, and accuracy were computed based on the generated confusion matrix for multi-class classification on the test set using the optimal set of hyperparameters. The performance scores are reported with 95% confidence intervals (CI), assuming a Gaussian distribution for the population. In terms of accuracy, the score tended to increase with the number of features. For the 150-feature set, the decision tree, random forest, and GBM algorithms achieved accuracy values of 92% (95% CI, 88–96%), 93% (95% CI, 89–97%), and 95% (95% CI, 92–98%), respectively. Our evaluation results indicate that feature sets with either 100 or 150 features outperformed sets with differing feature counts. Since the performance of the algorithm using the feature-selection methods was nearly identical to that using features selected by clinicians, we conclude that algorithms utilizing the appropriate number of features demonstrate high reliability.

**Table 5:** Comparison of the ML performance after feature selection

Number of features	Decision Tree				Random Forest				GBM			
	Precision	Recall	F-1 score	Accuracy	Precision	Recall	F-1 score	Accuracy	Precision	Recall	F-1 score	Accuracy
20	0.80$\:\pm\:$0.06	0.69$\:\pm\:$0.07	0.70$\:\pm\:$0.07	0.88$\:\pm\:$0.05	0.80$\:\pm\:$0.06	0.72$\:\pm\:$0.06	0.73$\:\pm\:$0.06	0.91$\:\pm\:$0.04	0.84$\:\pm\:$0.05	0.74$\:\pm\:$0.06	0.76$\:\pm\:$0.06	0.90$\:\pm\:$0.06
50	0.84$\:\pm\:$0.05	0.70$\:\pm\:$0.07	0.70$\:\pm\:$0.07	0.89$\:\pm\:$0.04	0.80$\:\pm\:$0.06	0.76$\:\pm\:$0.06	0.77$\:\pm\:$0.06	0.92$\:\pm\:$0.04	0.85$\:\pm\:$0.05	0.78$\:\pm\:$0.06	0.80$\:\pm\:$0.06	0.92$\:\pm\:$0.04
75	0.76$\:\pm\:$0.06	0.72$\:\pm\:$0.06	0.73$\:\pm\:$0.06	0.91$\:\pm\:$0.04	0.87$\:\pm\:$0.05	0.78$\:\pm\:$0.06	0.81$\:\pm\:$0.06	0.94$\:\pm\:$0.04	0.87$\:\pm\:$0.05	0.81$\:\pm\:$0.06	0.83$\:\pm\:$0.05	0.94$\:\pm\:$0.04
100	0.85$\:\pm\:$0.05	0.75$\:\pm\:$0.06	0.77$\:\pm\:$0.06	0.91$\:\pm\:$0.04	0.89$\:\pm\:$0.04	0.78$\:\pm\:$0.06	0.81$\:\pm\:$0.06	0.93$\:\pm\:$0.04	0.86$\:\pm\:$0.05	0.79$\:\pm\:$0.06	0.81$\:\pm\:$0.06	0.95$\:\pm\:$0.03
150	0.80$\:\pm\:$0.0	0.76$\:\pm\:$0.06	0.77$\:\pm\:$0.06	0.92$\:\pm\:$0.04	0.90$\:\pm\:$0.04	0.80$\:\pm\:$0.06	0.83$\:\pm\:$0.05	0.93$\:\pm\:$0.04	0.84$\:\pm\:$0.05	0.78$\:\pm\:$0.06	0.80$\:\pm\:$0.06	0.95$\:\pm\:$0.03
All	0.76$\:\pm\:$0.06	0.73$\:\pm\:$0.06	0.74$\:\pm\:$0.06	0.95$\:\pm\:$0.03	0.86$\:\pm\:$0.05	0.73$\:\pm\:$0.06	0.76$\:\pm\:$0.06	0.86$\:\pm\:$0.05	0.87$\:\pm\:$0.05	0.80$\:\pm\:$0.06	0.82$\:\pm\:$0.05	0.90$\:\pm\:$0.03
Expert50	0.92$\:\pm\:$0.04	0.71$\:\pm\:$0.07	0.72$\:\pm\:$0.06	0.91$\:\pm\:$0.04	0.90$\:\pm\:$0.04	0.77$\:\pm\:$0.06	0.80$\:\pm\:$0.06	0.94$\:\pm\:$0.04	0.84$\:\pm\:$0.05	0.76$\:\pm\:$0.06	0.78$\:\pm\:$0.06	0.93$\:\pm\:$0.04

**Fig. 3:**
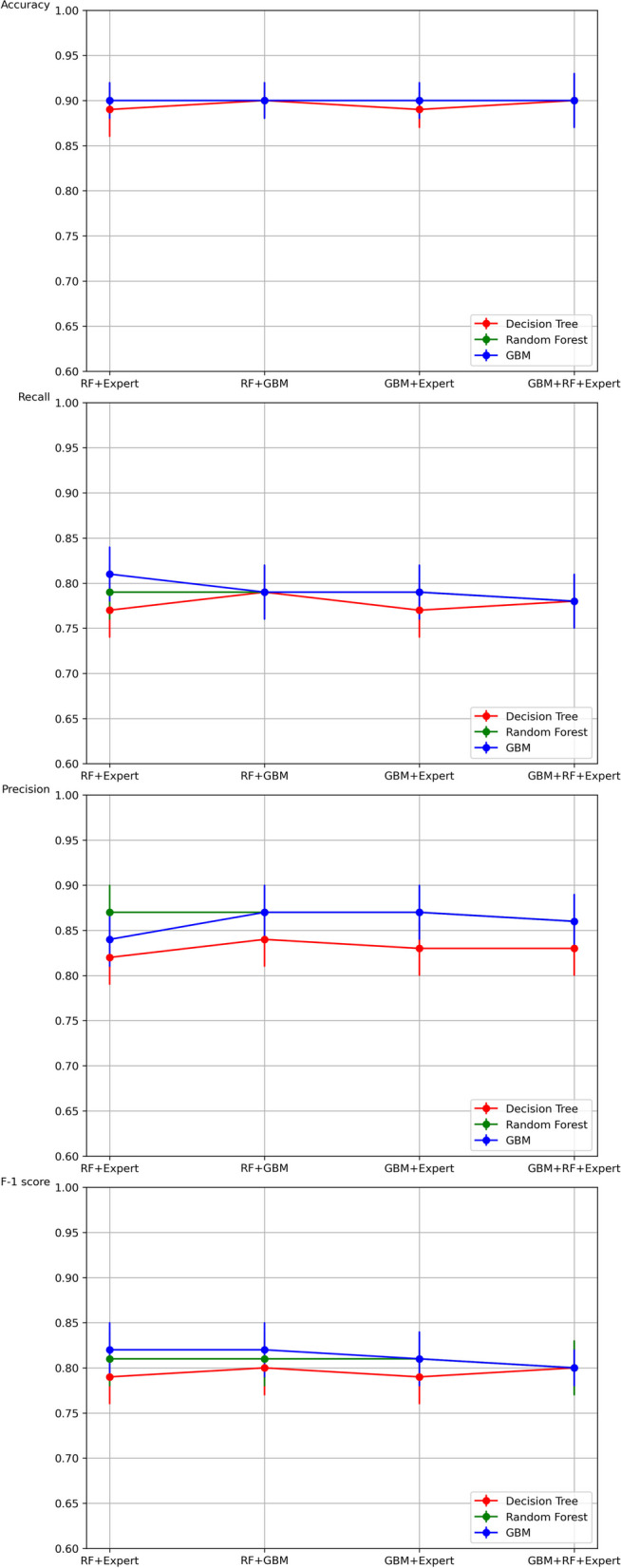
Comparison of the decision tree, random forest and gradient boosting machine performance with combined feature sets from different algorithms. (1) The combination of RF-selected features with expert-selected features. (2) Combination of RF features with GBM features, (3) Combination of GBM features with expert features, and (4) Combination of GBM, RF, and expert features

However, the primary objective of this study is to maximize accuracy to assist clinicians in their treatment decision-making process. Hence, rather than relying exclusively on a single feature selection method like RF, we employed multiple feature selection strategies to enhance accuracy. Specifically, we combined RF selected features with expert selected features (100 features), RF features with GBM features (100 features), GBM features with expert features (100 features), and GBM, RF, and expert features (150 features). Subsequently, we conducted DT, RF, and GBM algorithms using these four combined feature sets as inputs using optimal parameters. The results of this analysis are presented in Table 6; Fig. 3. However, we found that the results did not show any significant improvement.

**Table 6:** Comparison of the ML performance with combined feature sets

	Decision Tree				Random Forest				GBM			
	Precision	Recall	F-1 score	Accuracy	Precision	Recall	F-1 score	Accuracy	Precision	Recall	F-1 score	Accuracy
RF + Expert	0.82$\:\pm\:$0.03	0.77$\:\pm\:$0.03	0.79$\:\pm\:$0.03	0.89$\:\pm\:$0.03	0.87$\:\pm\:$0.03	0.79$\:\pm\:$0.03	0.81$\:\pm\:$0.03	0.90$\:\pm\:$0.02	0.84$\:\pm\:$0.03	0.81$\:\pm\:$0.03	0.82$\:\pm\:$0.03	0.90$\:\pm\:$0.02
RF + GBM	0.84$\:\pm\:$0.03	0.79$\:\pm\:$0.03	0.80$\:\pm\:$0.03	0.90$\:\pm\:$0.02	0.87$\:\pm\:$0.03	0.79$\:\pm\:$0.03	0.81$\:\pm\:$0.03	0.90$\:\pm\:$0.02	0.85$\:\pm\:$0.03	0.80$\:\pm\:$0.03	0.82$\:\pm\:$0.03	0.90$\:\pm\:$0.02
GBM + Expert	0.83$\:\pm\:$0.03	0.77$\:\pm\:$0.03	0.79$\:\pm\:$0.03	0.89$\:\pm\:$0.03	0.87$\:\pm\:$0.03	0.79$\:\pm\:$0.03	0.81$\:\pm\:$0.03	0.90$\:\pm\:$0.02	0.86$\:\pm\:$0.03	0.81$\:\pm\:$0.03	0.83$\:\pm\:$0.03	0.91$\:\pm\:$0.02
GBM + RF + Expert	0.83$\:\pm\:$0.03	0.78$\:\pm\:$0.03	0.80$\:\pm\:$0.03	0.90$\:\pm\:$0.03	0.86$\:\pm\:$0.03	0.78$\:\pm\:$0.03	0.80$\:\pm\:$0.03	0.90$\:\pm\:$0.03	0.84$\:\pm\:$0.03	0.81$\:\pm\:$0.03	0.82$\:\pm\:$0.03	0.90$\:\pm\:$0.02

**Fig. 4:**
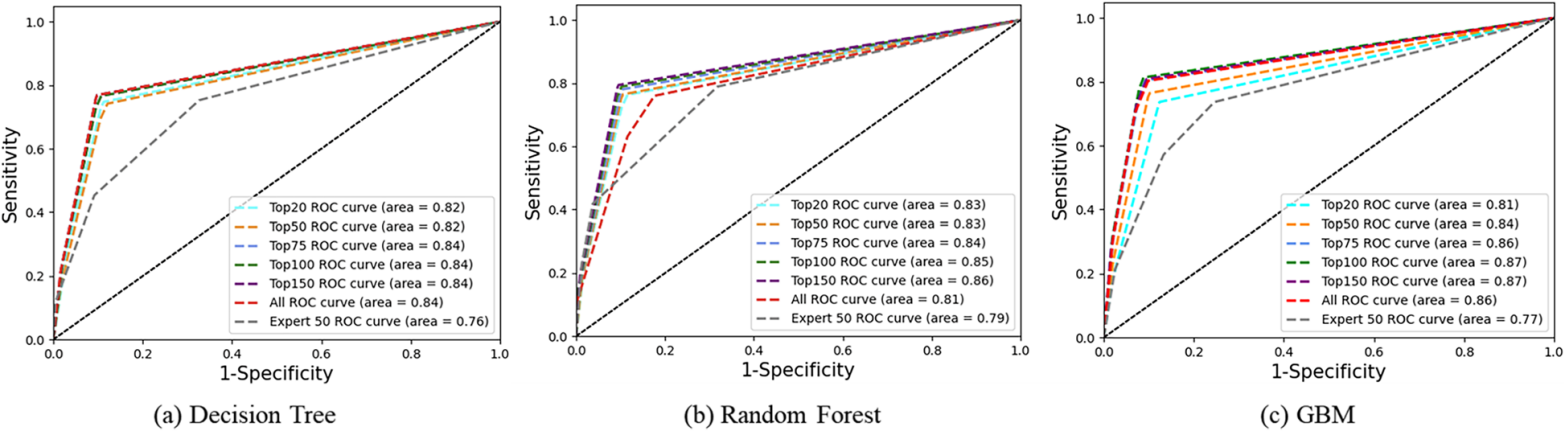
The ROC curves indicating the overall performance of the machine algorithms. The Gradient Boosting Machine has an AUC of 0.87

Finally, we analyzed the receiver operating characteristic (ROC) curves to assess the overall performance of the algorithms across different feature sets. As shown in Fig. 4, the GBM algorithm, when performed with either 100 or 150 features, achieved the highest area under the curve (AUC) of 0.87. In conclusion, a TDSS that employs ensemble ML algorithms and incorporates more than 100 features from real-world medical data is consistent with a clinician’s decision-making process for RCC treatment methods. These results highlight the feasibility and potential use of the proposed system.

## Discussion

This preliminary study aims to enhance the challenging medical environment by facilitating seamless and precise patient-specific treatment methods through a CDSS leveraging by big data and AI. To this end, we constructed RCC-related big data using information from a CDM—structured data designed for multicenter studies—and electronic medical records, which contain unconventional clinical data. Our exploratory efforts to develop a personalized TDSS focus on two primary objectives: to assist clinicians in determining standardized and optimal treatment protocols across diverse clinical situations, and to support individualized treatment decisions tailored to each patient.

Currently, the majority of AI research associated with RCC is based on radiomics using CT images, prognosis prediction models using ML or deep learning, and studies using messenger ribonucleic acid (RNA) and micro-RNA panels from The Cancer Genome Atlas. However, the literature contains no studies related to personalized TDSSs in the real world [28–31].

This research is a pilot study on a TDSS for RCC using ML algorithms. For the applicability, feasibility, and performance evaluation of the system based on CT readings, which are the most important points of assessment for disease diagnosis, a urologic oncologist at a single center divided the treatment methods into three groups by simplifying them. This classification was based only on CT readings and may differ from the treatments administered to patients in practice and standard guidelines. This is because, when deciding a treatment method in clinical research, the treatment method is ultimately decided considering various clinical scenarios (tumor location, diameter, shape, presence or absence of thrombosis, metastasis, general abdominal condition, and adhesion with adjacent organs on CT images, laboratory results related to RCC, International Metastatic RCC Database Consortium criteria, history of previous abdominal surgeries, patient preference, and cancer insurance, etc.). In a follow-up study, we intend to construct multicenter RCC big data and upgrade the development system in a situation similar to the real world by considering more diverse clinical scenarios.

The top ten features obtained by performing feature selection were consistent with the significant clinical features of RCC, which are considered the most important features by urologic oncologists when deciding the treatment plans in practice (Table 3). Among them, the top five features were the diagnosis name (RCC) and TNM clinical stage in the CT readings, which are the most important factors for determining the prognosis for patients with RCC in practice. In particular, the lymph node (N) plays a crucial role in deciding whether a patient should undergo surgical treatment or chemotherapy. Moreover, the selected features ranked 6th to 10th were primarily laboratory test indicators related to RCC. Alkaline phosphatase (ALP) acts as a marker enzyme of the brush border membrane of renal proximal tubular cells. Notably, a diminished ALP activity in RCC patients correlates with decreased cancer-specific survival rates. Metrics like the albumin content to ALP ratio and lipid profiles also emerge as unfavorable prognostic indicators for RCC [32–36]. Both hemoglobin and hematocrit serve as vital determinants in choosing the appropriate surgical treatment method and in prognostic evaluations, as highlighted in the treatment guidelines for advanced RCC patients [8]. LDH, a glycolytic enzyme, facilitates the transformation of pyruvate into lactate, potentially holding significance in tumor metabolism [37]. Serum LDH levels stand out as key biomarkers in assessing the prognosis and oncological outcomes of RCC [40]. The performance evaluation of models based on the Expert50 feature set closely resembled those trained after selecting features from the feature set. Therefore, we suggest that our RCC-Supporter can be integrated into the TDSS for RCC in a clinical setting.

However, this study presents several limitations and concerns. Firstly, the dataset comprises only structured and unstructured data from a single institution, potentially leading to a limited patient count for the development of AI algorithms. Nonetheless, as we are in the process of aggregating multicenter data, this approach is anticipated to be scalable to extensive big data in future phases. Secondly, while the current model uses textual data for pathological results and CT readings, future iterations aim to incorporate pathologic slide images and CT scans to enhance the comprehensiveness and sophistication of the CDSS. Thirdly, an exploration into cases where the model made incorrect predictions would be highly informative. The original treatment groups classified in this study and the treatment groups predicted by a machine learning algorithm using expert-selected features were confirmed, and a total of 166 patients (8.9%) were classified differently. When the predicted factors were analyzed through multivariate analysis, the representative factors were age (OR 1.243 [1.037–1.490], *P* = 0.018) and T_nan (OR 7.324 [3.762–14.255], *P* < 0.001). In the clinical real world, although surgical treatment is possible for elderly patients, they are often subjected to active surveillance or observation due to the risks of surgery. Additionally, even though elderly patients may need chemotherapy, they are often unable to receive it or refuse it. In the case of the T_nan factor, it can be said to be the most significant factor in deciding treatment. This study analyzed CT reading text files. Since there were cases where the specific T stage was not mentioned in the reading, a more accurate system could be implemented if correction was made for this. In addition, treatment groups were classified based on a urologist’s judgment by only looking at the collected data from a single institution, and this was compared and analyzed with a machine learning algorithm. This may differ from the actual treatment these patients received. The prediction and performance of the model can be improved by using big data from multiple institutions and including a larger amount of clinical data, including radiologic images, pathologic slides, clinical history, and clinical manifestations. Lastly, it’s important to note that actual patient treatments may differ from the treatment scenarios considered in this study. The classification of each patient’s treatment was based on international guidelines as closely as possible using the available data. However, in an actual clinical environment, different treatments are often selected for each patient than those suggested in the guidelines due to various clinical situations. For example, for a patient with a 3 cm small RCC mass in the right kidney, partial nephrectomy is recommended according to the guidelines. However, even for this small mass, radical nephrectomy can be performed if it is adjacent to surrounding major vessels or due to underlying disease. Therefore, to develop a more sophisticated system, comparative analysis of treatment using this system and actual treatment received by patients, and further research using real-world data from multiple centers and that covers diverse clinical conditions is essential.

## Conclusions

In this study, real-world medical big data on renal cell carcinoma patients were collected from a single center. Based on this big data, a preliminary treatment decision-support system (TDSS) was developed for the first time by applying well-known machine learning algorithms such as Decision Tree, Random Forest, and Gradient Boosting Machine. This system aims to provide personalized treatment to overcome the difficulties in treatment selection according to various individual clinical situations, and its feasibility has been confirmed. In the future, we plan to enhance the system by incorporating multi-center clinical big data to consider more complex clinical situations and to validate its applicability in real clinical settings.

## Data Availability

The datasets generated and/or analyzed during the current study are not publicly available due to the hospital policy but are available from the corresponding author on reasonable request.

## Abbreviations

ALP: Alkaline phosphatase
AUC: Area under the curve
CDM: Common data model
CDSS: Clinical decision support system
CT: Computed tomography
DT: Decision Tree
EHR: Electronic health record
GBM: Gradient boosting machine
LDH: Lactate dehydrogenase
MICE: Multivariate imputations by chained equations
OMOP: Observational Medical Outcomes Partnership
RF: Random Forest
RCC: Renal cell carcinoma
ROC: Receiver operating characteristics
RWD: Real-world data
TDSS: Treatment decision support system
TNM: Tumor (T), node (N), and metastasis (M)
ICD: 10 International Classification of Diseases 10th Revision
SNOMED-CT: Systematized Nomenclature of Medicine-Clinical Terms
